# Evaluating scar outcomes in pediatric burn patients following skin grafting

**DOI:** 10.1038/s41598-025-06378-y

**Published:** 2025-06-20

**Authors:** Ingrid Steinvall, Sharon Kennedy, Matilda Karlsson, Mohamed A. Ellabban, Folke Sjöberg, Caroline Andersson, Moustafa Elmasry, Islam Abdelrahman

**Affiliations:** 1 Department of Hand Surgery, Plastic Surgery and Burns, Department of Biomedical and Clinical Sciences, Linköping University, 58185 Linköping, Sweden; 2 Department of Plastic, Reconstructive and Aesthetic Surgery, Royal College of Surgeons in Ireland, Dublin, Ireland; 3 Department of Plastic and Reconstructive Surgery, Faculty of Medicine, Suez Canal University, Ismailia, Egypt; 4 Department of Anaesthesiology and Intensive Care, Linköping University, Linköping, Sweden

**Keywords:** Burns, Children, Healing time, Scar quality, Skin graft operation, The patient and observer Scar assessment scale, Medical research, Outcomes research

## Abstract

Scarring and its long-term sequelae, contribute significantly to morbidity following burn injuries. Factors associated with less favourable scar outcomes include the depth of burn, younger age, pigmented skin types and prolonged healing times. The aim of primary burn surgery is to debride non-viable tissue, to enable healing. However, international consensus regarding the optimal timing for debridement and grafting in pediatric patients with burns is lacking. Delayed wound healing is thought to increase the risk of poor scar quality, however, the evidence for this is weak with few studies investigating long-term outcomes in pediatric patients. The aim of this study, therefore, was to investigate the effect of patient and treatment factors on scar quality, one year after skin grafting in pediatric patients with burns. Patient factors included age, skin type, and site of burn, while treatment factors included timing of surgery, type of surgery, and healing times. Pediatric patients (age < 18 years) presenting to a National Burn Unit from 2011 until 2020, inclusive were considered for inclusion in the study. Burn injuries between 1% and 14.9% total body surface area (TBSA) and who required skin grafting for the primary treatment of their burn, were included. Patients who failed to attend their 12-month follow-up visit were excluded. Standardised clinical photographs were assessed using a modified version of the Patient and Observer Scar Assessment Scale, version 2.0 (POSAS). Thirty children (median age 3.9 years) were included. Factors with an independent effect on higher (worse) POSAS scores were younger age at the time of injury (*p* < 0.001), body site of the trunk (*p* < 0.002), or the lower extremity (*p* < 0.001) and a longer duration of healing time after skin grafting (*p* = 0.003). The duration of time between injury and surgery was not an independent factor for POSAS scores (*p* = 0.56). We had insufficient numbers to discriminate differences in scar quality for different graft types; meshed versus non-meshed. In this study, we found that long-term scar outcomes in pediatric burn patients after skin grafting were worse for those injured at a younger age, with burns on the trunk or lower extremity, or with prolonged healing time after grafting. The robustness of this conclusion is limited by the small sample size of the study cohort and by our use of photographic scar assessment .

**Supplementary Information:**

The online version contains supplementary material available at 10.1038/s41598-025-06378-y.

## Introduction

Poorer scar outcomes after burn injury are associated with larger, deeper burns, injury at a younger age, burns in pigmented skin types and a longer time to healing after injury. In particular, there is an increased risk of hypertrophic scarring with these factors^1–5^. While studies have addressed scar quality in pediatric populations after burns, long-term follow-up of outcomes are lacking^5–8^.

The timing of surgical intervention in pediatric populations with partial thickness burns, remains a topic of ongoing debate, with no established international consensus^9^. The decision between early debridement (withing 72 h) or debridement when the burn has fully demarcated (1–3 weeks) can be difficult. Accurate clinical assessment to correctly determine the depth of the burn is a key step in this process^10^. Underdebridement can result in prolonged wound healing leading to increased infection rates^11^ and poorer scar quality. However, the evidence linking delayed wound healing to poorer scar outcomes is limited and inconsistent^12^.

Accurate assessment of burn depth is difficult, even for experienced clinicians, with accuracy rates as low as 50–75%^11^. Many objective measures have been proposed to improve assessment accuracy, including histologic analysis^13^, laser doppler imaging (LDI)^14^, thermography^15^, thermal imaging^16^, photoacoustic imaging^17^, and fiber-optic confocal imaging^18^, to name but a few. All methods have advantages; however, there is strong evidence that LDI offers superior construct validity compared to other techniques^19^. Despite this, the routine use of LDI in the clinical setting remains inconsistent, and clinical assessment continues to be the most commonly used method^20^.

Evaluation of scar quality is a complex process that requires collaboration between the patient and a clinician experienced in scar evaluation. The process can be influenced by various factors. Beliefs and perceptions about the scar, the injury and the patient’s treatment can all influence opinion about an outcome. Observer- and patient-rated items are subjective in nature, which can introduce variability and reduce consistency across evaluations^21^. The Patient and Observer Scar Assessment Scale (POSAS) version 2.0, is a widely used and robust scar assessment tool, designed to address some of these challenges. The POSAS demonstrates good internal consistency and reliability for the observer scale, the overall score and for separate items of the scale^22^. It is a condition-specific outcome measure and has become a standard for evaluating burns scars^23,24^.

The aim of this study was to evaluate the impact of patient factors and treatment factors on long-term scar quality in pediatric burn patients, using the POSAS 2.0.

Patient factors included age, skin type and burn site, while treatment factors included the timing and type of surgery and healing duration after grafting.

## Methods

In this retrospective cohort study, consecutively referred children treated at a National Burn Unit from 2011 to 2020, inclusive, and who sustained burn injuries requiring split-thickness skin grafting during their acute management, were considered for inclusion.

**Fig. 1:**
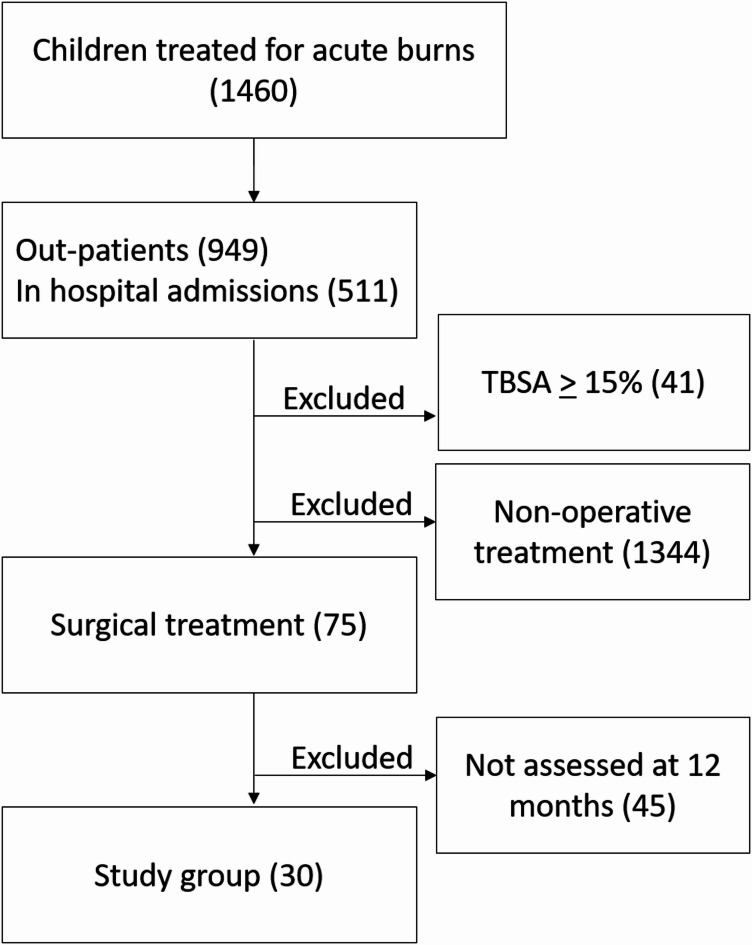
The flowchart shows patient selection from all children managed at the Burn Centre during the period 2009–2020. Of the total number (1460) 511 were in-hospital admissions and 949 were treated at the out-patient clinic.

Patients younger than 18 years with a burn size between 1% and 14.9% total body surface area (TBSA) as determined by consensus between two senior clinicians with over 10 years of burns care experience, (IA, ME), were eligible for inclusion. The size and depth of the burn was assessed clinically during initial evaluation, either on admission to the hospital or on first presentation to the outpatient clinic. Each burn was evaluated for color, capillary refill and sensory changes in order to clinically determine the depth of injury. The size of the burn, its distribution and assessed depth were recorded on a Lund and Browder chart. Burn size was estimated using the patient’s own palm size and confirmed upon completion of the Lund and Browder chart through secondary review of clinical photographs. Patients with burn sizes of 15% TBSA or greater were excluded, as the systemic inflammatory response in larger burns may significantly impact healing and scar formation, introducing additional confounders. We also excluded children who underwent dermal reconstruction with a dermal substitute prior to skin grafting, as their scars may differ from those associated with skin grafts alone. Additionally, we excluded patients who did not require skin grafting and those who did not attend for scar assessment 12 months post-surgery (Fig. 1).

Debridement and skin grafting were performed under general anaesthetic, by at least one senior surgeon (IA, ME). The Zimmer^®^ Air Dermatome was used to harvest the skin for grafting. The typical settings for skin harvest were 10/1000 inch, with every effort to take thin skin grafts to avoid donor site morbidity. Grafts were either meshed or applied as unmeshed split-thickness grafts. The decision to apply meshed or unmeshed grafts was made by a senior surgeon (IA or ME), however, this decision followed the standard practice in our clinic. In general, unmeshed grafts are used for cosmetically sensitive areas such as the face, hands and neck. Unmeshed grafts are also used if the defect size is small (1–2%). All other wounds are usually reconstructed with meshed grafts. All meshed skin grafts were meshed with a ratio of 1.5:1.

Patients underwent post-operative scar evaluation by an occupational therapist, experienced in scar management. Scar treatment was comprised of prophylactic compression garments (10–18 mmHg), commenced as soon as the skin was sufficiently healed. The burn wound was considered sufficiently healed if 90% of it was epithelised. If deemed necessary by either a senior surgeon (IA, ME) or by the treating occupational therapist, tailored therapeutic compression garments (20–30 mmHg), were prescribed. The indications included hypertrophic scars, scars crossing joint lines or larger confluent burn scars. When used, therapeutic compression was commenced within one to three months post-operatively. Patients were encouraged to wear garments for as many hours as possible each day, but for at least 12 h. Additional adjuncts of silicone gel sheets, silicone foam dressings or silicone gel were prescribed as deemed necessary by the treating senior doctor or scar occupational therapist. Intra-lesional injections of corticosteroids were prescribed for more severe hypertrophic scarring. Scar treatment was considered complete when the scar was soft, supple and asymptomatic or the scar was greater than 18 months post healing, whichever was longer. We deliberately allowed scar therapy to be individualised to each patient, rather than following a protocol. This was to mirror normal clinical practise. We also wished to avoid either over treating or depriving pediatric patients of required scar interventions, which we considered as unethical.

**Fig. 2:**
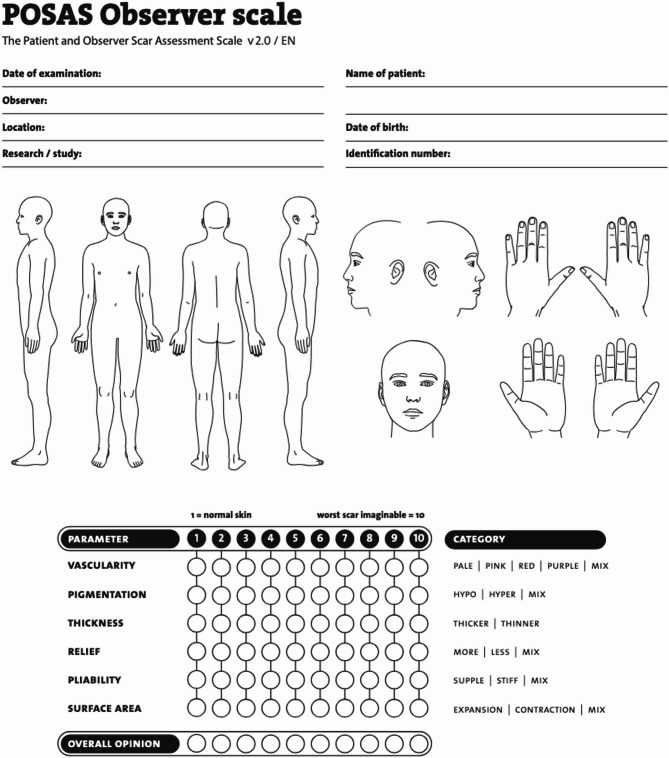
The POSAS 2.0 Observer Scale.

**Fig. 3:**
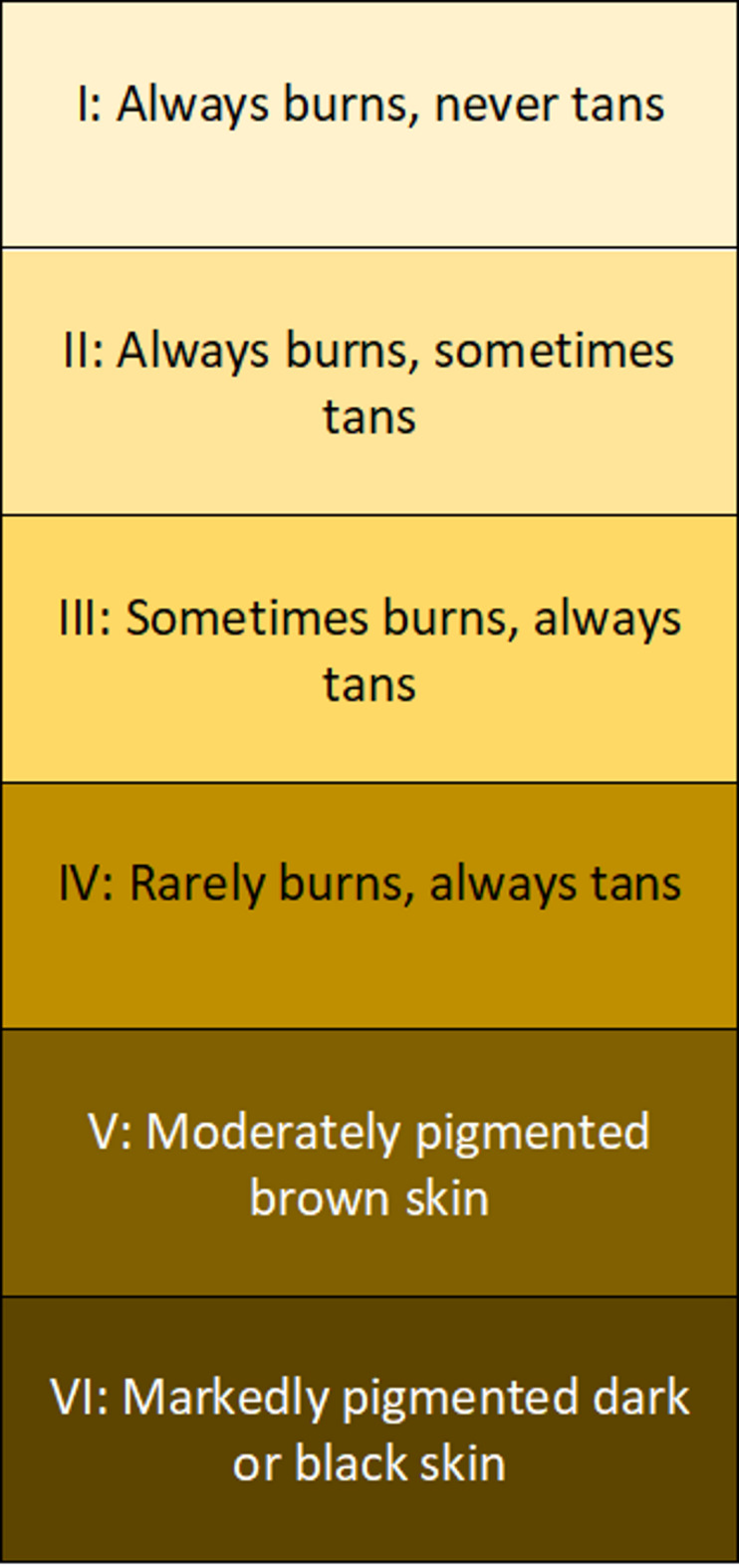
Fitzpatrick skin type classification.

Clinical information and demographic data were retrieved from patients’ medical records. Clinical photographs were taken in a standardised format. Photographs were taken in the same well-lit room in the burn out-patient department. All burns wounds were undressed, cleaned and dried prior to being photographed. Images were taken at a 90-degree angle to the scar from a distance of approximately 10–15 cm, with a second image taken from a distance of approximately 40 cm. Images taken through-out the study were obtained with the same camera and viewed on a network computer, within the hospital, which all have the same display settings. Scar assessment comprised of the observer portion of the Patient and Observer Scar Assessment Scale (POSAS 2.0)^23,25^. The same senior occupational therapist, with greater than 10 years’ experience in assessing burn scars, reviewed photographs of the grafted areas and completed the assessments. Although not originally designed for scoring via photographs, there is some evidence that this is a valid method of utilising POSAS 2.0^26^. Images were taken at 3- and 12-month intervals post-operatively and were assessed in the same way at both time points. Although enrolment in the study was prospective, the scar assessments by the senior occupational therapist occurred at the end of the study period. Once all 3- and 12- month images had been obtained, the assessments were then completed. The assessor was blinded to the timing of the surgery (i.e. whether it was a 3- month or a 12- month photograph), overall healing time, scar treatments received and patient identification. The items scored on the POSAS 2.0 (Fig. 2) were vascularity, pigmentation, thickness, relief and surface area. The item ‘pliability’ was not assessed and therefore excluded, as palpation did not form part of the assessment^26^. For each item, the scores ranged from 1 to 10, with 1 indicating the best possible outcome and 10 indicating the worst outcome. The POSAS score was calculated as the sum of individual item scores, with a minimum possible score of 5 representing the best outcome and a maximum score of 50 indicating the worst outcome. Each item on the POSAS includes descriptive categories to further characterize the scar. For example, vascularity can be categorised as pale, pink, red, purple, or mixed. Pigmentation is described as hypopigmentation, hyperpigmentation, or mixed pigmentation. Thickness is classified as either thicker or thinner compared to normal skin. Relief is described as having more relief, less relief, or mixed. Finally, surface area is categorized as expanded relative to the original area, contracted, or mixed. Skin type was classified according to the Fitzpatrick scale (Fig. 3) and comprises of six skin types as follows: (I) always burns, never tans; (II) always burns, sometimes tans; (III) sometimes burns, always tans; (IV) rarely burns, always tans; (V) moderately pigmented brown skin; (VI) markedly pigmented dark or black skin^27^.

### Statistics

The distribution of data was evaluated using the Lilliefors test. Results are presented as median (25–75th percentile) or n (%), unless otherwise specified. A *p*-value of < 0.05 was considered statistically significant. Group differences were analysed using the Mann Whitney U test, Kruskal-Wallis ANOVA, or chi-squared tests (or Fisher’s exact tests), as appropriate. The Wilcoxon matched-pairs test was employed for intra-individual comparisons. Multivariable linear regression was used to evaluate the effects of age, sex (male = 1, female = 0), skin type, burn size (TBSA %), size of deep dermal and full thickness burns, skin grafted areas, body site (upper extremity, lower extremity, trunk, head and neck), burn type (scald burn, contact burn, flame burn), timing of the operation (days from injury to skin grafting), multiple surgeries (yes = 1, no = 0), total healing time, healing time post-surgery and antibiotic treatment pre and post-surgery (yes = 1, no = 0), on the POSAS score at the 12-month assessment.

Manual stepwise forward selection was applied, retaining variables that significantly contributed to the model (substantial increase in R^2^ and p values < 0.05). Additionally, “skin grafted area”, “days from injury to operation” and “skin type” were included due to their relevance. No corrective measures were applied for skewed variables in the regression. The presence of heteroscedasticity was evaluated using the Breusch-Pagan test and multicollinearity was assessed through the variance inflation factor (VIF).

### Ethics approval

All methods were conducted in accordance with relevant guidelines and regulations. The study received approval from the Swedish Ethical Review Authority (No. 2021–04598). No experimental protocols were employed, and results were not presented at the individual level to protect the confidentiality of the study participants. Due to the observational nature of the study, the requirement for obtaining informed consent was waived by the Swedish Ethical Review Authority.

## Results

Thirty children were included in the study. The median age was 3.9 years (1.5–11.6). Of these, 16 (53%) were boys and 17 (57%) had Fitzpatrick skin type II. Scald burns were the most common mechanism of injury (Table 1). The median TBSA of burns was 4.8%. The median skin grafted area was 0.8% TBSA (25th – 75th centile 0.5–1.1). The TBSA requiring grafting was slightly smaller than the area of burns as assessed on admission, however, this was not significant (*p* = 0.11). All grafts were split thickness grafts. Grafts were meshed in 20 (67%) patients. The remainder were unmeshed grafts. Patient and burns characteristics are presented in Table 1.

**Table 1:** – Details of the patients.

Age, years	3.9 (1.5–11.6)
Male sex	16 (53)
Skin type:	
I	5 (17)
II	17 (57)
III	6 (20)
IV	1 (3)
V	1 (3)
Burn type:	
Scald	20 (67)
Contact burn	6 (20)
Flame burn	4 (13.3)
Burn size, TBSA%	4.8 (0.8–7.1)
Superficial dermal burn, TBSA%	2.8 (0.0–5.1)
Deep dermal burn, TBSA%	0.9 (0.1–2.0)
Full thickness burn, TBSA%	0.0 (0.0–0.0)
Deep dermal and full thickness burn, TBSA%	1.0 (0.3–2.1)
Operated and skin grafted area, TBSA%	0.8 (0.5–1.1)
Meshed skin graft	20 (67)
Days from injury to operation	13.5 (9.8–15.5)
Patients with two operations	4 (13)
Antibiotic administration (before operation)	16 (53)
Antibiotic administration (after operation)	12 (40)
Total healing time, days from injury	28.0 (25.5–40.0)
Days from operation to healing	14.0 (12.8–28.8)

Data are presented as median (25th − 75th centile) and n (%). Skin type classified according to the Fitzpatrick scale. TBSA, total body surface area. Deep dermal and full thickness burn = deep burns that may require surgery (the sum of deep dermal and full thickness burn TBSA%). *n* = 30

### Scar assessment over time

The median time from burn injury to surgery was 13.5 days (25–75th centile 9.8–15.5). The first follow-up assessments occurred 3.6 months after injury (25–75th centile 3.1–4.3) and 3.2 months after skin grafting (25–75th centile 2.5–3.7). The second follow-up assessments were conducted 12.4 months after injury (25–75th centile 12.1–12.9) and 12.0 months after skin grafting (11.7–12.5).

POSAS observer scores decreased (improved) by a median of 5 points between the 3- and 12-month assessments. The median POSAS score was 17 (25–75th centile 14–19.3) at the 12-month assessment. All items showed a median decrease of 1 point except for ‘relief’, which remained unchanged (Table 2).

All scars rated as red at three months, had changed to pink or pale by the 12-month assessment. There was a tendency towards a reduction in hyperpigmentation and an increase in hypopigmentation, although a third of the scars were assessed as having mixed pigmentation. The proportion of scars assessed as thick was unchanged. Similarly, the proportions in the relief and surface area categories remained unchanged between the 3-month and 12-month assessments. (Supplemental Table S1).

### POSAS results at 12 months by timing of surgery, graft type, and Fitzpatrick skin type

Subgroup analysis by timing of surgery (early skin grafting < 14 days after injury versus late skin grafting  ≥ 14 days after injury) showed lower POSAS scores in the ‘vascularity’ and ‘thickness’ categories in pediatric patients who underwent grafting  ≥ 14 days after injury compared to < 14 days after injury (p-values 0.02 and 0.01) (Supplemental Table S2). When graft type was considered, pediatric patients who underwent grafting with unmeshed grafts had lower POSAS scores in the ‘relief’ category compared to those with meshed grafts. This difference was borderline significant (*p* = 0.049) (Supplemental Table S3). Analysis by Fitzpatrick skin type showed no significant differences in POSAS scores between groups. Post hoc analysis revealed a trend (*p* = 0.09) toward lower scores for skin type I compared with skin types III, IV, and V on the POSAS item ‘pigmentation’ (Supplemental Table S4).

### Multivariable analysis of POSAS scores at 12 months

Higher (worse) POSAS scores were significantly associated with younger age, longer postoperative healing duration and skin grafts located on the trunk and lower extremities, compared to grafts on the upper extremities (or the head and neck as tested in an alternative regression model). The coefficients showed that, after adjusting for other factors, for each year younger in age, the mean increase in POSAS score was 0.7 points (95% CI 1.0–0.3). For each additional day of healing following surgery, the mean increase in POSAS score was 0.2 points (95% CI 0.1–0.3). Skin grafting on the trunk was associated with a mean increase in POSAS score of 7.8 points (95% CI 3.1–12.4) and skin grafting on the lower extremity was associated with a mean increase of 11.2 points (95% CI 6.6–15.8). The type of skin graft applied, meshed or unmeshed, did not contribute meaningfully to the regression analysis. We considered that this was likely due to insufficient numbers to allow for reliable discrimination between these two cohorts. Neither the size of the skin graft nor the timing of surgery had independent effects on the POSAS score after adjusting for other factors. The remaining demographic and burn characteristic variables, presented in Table 1, were assessed along with POSAS scores at three months; however, none significantly contributed to the final model and were therefore, omitted.

### POSAS results at 12 months by body site

POSAS results (Supplemental Table S2) showed a disadvantageous effect of early surgery, which was not observed after adjusting for body site (Table 3). This suggests a potential selection bias related to the timing of surgery and anatomical site of injury. POSAS scores were higher for the trunk and lower extremity compared to the upper extremity and head and neck regions (Supplemental Table S5). However, since the upper extremity was the most common site for later surgery, differences in group sizes may have influenced these findings. Supplemental Table S6 indicates that the POSAS item ‘surface area’ (*p* = 0.03) primarily accounted for differences in POSAS scores between body sites, with the highest surface area values reported on the lower extremity and trunk.

**Table 2:** Scar quality over time, POSAS Observer score values by each item and score sum at the three- and 12-months’ follow up.

	3 months	12 months	*p* value
Vascularity	3.0 (2.8–4.0)	2.0 (1.0–2.0)	< 0.001
Pigmentation	3.0 (2.8–4.3)	2.5 (2.0–3.0)	0.02
Thickness	3.0 (2.0–5.0)	2.0 (2.0–3.0)	0.007
Relief	3.0 (2.8–4.0)	3.0 (2.8–4.0)	0.13
Surface area	8.0 (7.0–9.0)	7.0 (5.0–8.0)	0.001
POSAS score sum	22.0 (18.8–24.0)	17.0 (14.0–19.3)	< 0.001
Months after injury	3.6 (3.1–4.3)	12.4 (12.1–12.9)	< 0.001
Months after skin graft	3.2 (2.5–3.7)	12.0 (11.7–12.5)	< 0.001

Data are presented as median (25th – 75th centile), *n* = 30. Wilcoxon Matched Pairs Test.

**Table 3:** Multivariable linear regression for the association between POSAS score sum 12 months after operation and the variables that contributed significantly to the model and those that were kept because of interest.

	Coefficient	*p* value	95% CI low	95% CI high
Age, years	– 0.7	< 0.001	– 1.0	– 0.3
Operated and skin grafted area, TBSA%	– 0.3	0.34	– 1.0	0.4
Days from injury to operation	0.1	0.56	– 0.2	0.3
Days from operation to healing	0.2	0.003	0.1	0.3
Body site:				
Upper extremity (reference)				
Trunk	7.8	0.002	3.1	12.4
Lower extremity	11.2	< 0.001	6.6	15.8
Head and neck	– 0.4	0.86	– 4.3	3.6
Skin type:				
I (reference)				
II	– 1.5	0.41	– 5.1	2.1
III–V	0.8	0.70	– 3.2	4.7
Constant	14.2	< 0.001	9.4	18.9

TBSA, total body surface area. Model *p* < 0.001, adjusted R^2^ 0.59, *n* = 30. Skin types III (*n* = 6), IV (*n* = 1), and V (*n* = 1) were grouped because of small numbers. The Breusch-Pagan test showed *p* = 0.58 and the variance inflation factor analysis showed VIF values below 4.

## Discussion

Our study examined the long-term scar quality following skin grafting in pediatric patients with burns. Younger age, grafts to the trunk and lower extremities, and a longer duration of healing after surgery independently impacted POSAS scores one-year after injury. The relatively high R^2^ value of the regression model reinforces the robustness of these findings.

Younger age was identified as an independent factor associated with lower scar quality, as indicated by higher POSAS scores in our study. The risk of hypertrophic scarring has previously been reported to be higher in children than in adults^5,28^. However, more recent studies using the POSAS instrument have not found age to be an independent factor influencing scar quality^6^. Scar height has also been reported to be greater in younger children (under five years of age) compared to those aged five to eighteen years. However, this effect was not significant after adjusting for factors such as burn size, time to healing and multiple operations^3^. We believe that the use of different variables in regression analyses may account for some of the conflicting results observed between studies.

Scar progression can vary depending on its anatomical site. McDonald et al. assessed 60 skin grafts in 26 children under 14 years of age. They found that scars on the head, neck and buttocks were strongly associated (100%) with hypertrophic scarring (‘fair’ or ‘poor’ scar quality), followed by scars on the foot (60%), hands (50%), chest, upper extremity, back, and lower extremity (38–31%). In contrast, no hypertrophic scars were reported on the abdomen. The scale used in their study graded scars based on both the extent and height of hypertrophic scarring. ‘Fair’ was defined as more than 5% of the graft being elevated (thickened) by more than 1 mm, while ‘poor’ was defined as elevation greater than 2 mm or the presence of a contracture^5^. While differences in methods of assessment may account for some of the discrepancies between our study and that of McDonald et al.; we found that anatomical sites on the trunk and lower extremity independently influenced POSAS scores. This effect was primarily driven by higher values on the ‘surface area’ item (Supplemental Table S6).

This study was not designed to investigate cellular mechanisms underlying the association between factors such as younger age or specific graft locations and worse scar outcomes. However, it is worth considering potential mechanisms, as these factors have been previously linked with poorer scar outcomes^5,28^. Younger children differ from adults in that they tend to have a more pronounced inflammatory response. Additionally, children’s skin is characterised by a higher density of fibroblasts and increased collagen production, which may contribute to a greater tendency to form thickened or hypertrophic scars^29^. Scar formation varies across anatomical locations, with certain areas commonly associated with poorer scar quality. Regions under increased tension, such as periarticular skin, the neck, or the sternum, are particularly affected. This phenomenon is hypothesized to result from mechanical forces across the healing wound, which disrupts the normal wound healing cycle. Inflammation is prolonged, leading to increased production and differentiation of fibroblasts to myofibroblasts leading to scar thickening^30^. These factors often interplay to create both functional and aesthetic challenges.

The timing of surgical intervention (i.e., the duration between injury and skin grafting) did not independently effect POSAS scores in our study. These findings align with results reported by Ayaz et al. and Mohammadi et al., who observed no significant differences in scar quality between early (within the first two weeks) and delayed skin grafting. Scar quality was assessed in their study using the Vancouver Scar Scale six months postoperatively among both children and adults^12,31^. McDonald et al., in contrast, reported a higher risk of hypertrophic scar formation when skin grafting was performed later than 14 days post injury in a mixed cohort of children and adults^5^. However, the heterogeneity of their cohort may have influenced these findings, given the known differences in scar formation between adults and children.

The importance of achieving healing within three weeks to minimise hypertrophic scarring is well documented^1,2,32,33^. A linear relationship between prolonged healing times and hypertrophic scar formation has been observed in conservatively managed pediatric patients with burns^2,33^. Cubison et al. further reported that children with extended healing times (> 21 days) had a similarly high risk of hypertrophic scarring, regardless of whether they were treated conservatively or underwent skin grafting. Interestingly, children who healed within 14 days post grafting, demonstrated a higher risk of hypertrophic scarring than those who healed between 15 and 21 days. This may suggest that very rapid healing does not always confer better scar outcomes^2^. In line with these findings, our study identified both total and post-operative healing times, as independent factors contributing to higher POSAS scores. (Table 3).

Graft type was not associated with POSAS scores in simple linear regression. However, after adjusting for anatomical site in the multivariable model, meshed grafts were associated with lower POSAS scores. As mentioned above, the choice to use meshed or unmeshed grafts was made by a senior surgeon based on the patient and burn characteristics. This decision generally followed the standard practices of the clinic (detailed above). However, it is possible this may have introduced bias into the results and may explain why the anatomical site is a confounding variable. Given that the direction of the association was contrary to expectations, we propose that the result may be due to a combination of bias and insufficient power to accurately discriminate between meshed and unmeshed graft scars.

The association between Fitzpatrick skin type and the risk of hypertrophic scarring is well documented in the literature^1^, although the evidence remains limited when considering long-term scar outcomes in pediatric patients^1,28^. McDonald et al. found that a non-white skin type was associated with an increased risk of hypertrophic scarring among adults after skin grafting. However, the proportion of hypertrophic scarring among children with a non-white skin type (57%, 25/44 sites with “fair” or “poor” scar quality) was not significantly higher than in children with a white skin type (31%, 5/16 sites with “fair” or “poor” scar quality)^5^. Chipp et al. studied 383 children under 16 years of age. In their study, they found children with non-white skin types, and who were treated conservatively, had a tendency towards higher rates of hypertrophic scarring. However, these findings did not reach statistical significance despite the relatively large study group^33^. Wallace et al. investigated risk factors for hypertrophic scarring in a cohort of 186 children under 16 years of age. In their study, half were treated conservatively, while the other half underwent skin grafting. They could not establish skin type as an independent factor for hypertrophic scarring^3^. Our results indicate that regression analysis (Table 3) revealed no independent association between skin type and POSAS scores; however, this finding should be interpreted with caution given the limited number of children classified as having skin type III or higher.

The retrospective and single-centre design of our study, along with the relatively small sample size, represent limitations of this work. Although POSAS was not originally intended to be calculated from photographs, it has been used in this way in previous studies^34^. Additionally, the diverse geography of the study population made face-to-face follow-up assessment impractical. Although all photographs were assessed by the same senior occupational therapist, we believe this standardisation of the observer POSAS scores minimised inter-tester reliability discrepancies, which can often be problematic^34^. Nevertheless, it would may have been more robust to perform a prospective study in which all skin grafts were evaluated with the entire Patient and Observer POSAS at a 12-month visit.

Compression may be more effective over bony areas, such as the limbs and scalp, compared to the abdomen, for example. As scars were worse in our cohort at both anatomical sites of the lower limb and the trunk, it is difficult to ascertain whether the effectiveness of compression contributed to this outcome. Larger studies may be required to independently assess the effect of compression by body site.

One of the components of the POSAS is scar thickness, which we acknowledge is incompletely assessed via photographic assessment and may be subject to bias via clinical assessment. The addition of objective measures of scar thickness, such as high frequency ultrasound or histological assessment may be more robust.

Long-term scar outcomes can be affected by multiple variables. Prolonged healing times are one such variable and shown in this study to be associated with poorer scar outcomes. Graft take or graft infection rates were not looked at, as causes for prolonged healing in our study. This is a limitation as it is possible that infection or poor graft take may have had a greater influence on poor scar quality. These would be interesting variables to investigate in future work.

Scar treatment interventions such as compression, silicone therapy, and massage were not standardised in our cohort. This reflected routine clinical practice, whereby treatments were tailored to patients’ needs. The lack of standardisation, however, may have introduced bias in POSAS scores and/or masked confounding variables in the results. It is important, therefore, to interpret the findings in that context.

The distribution of Fitzpatrick skin types in our study is a limitation. The analysis of this variable may have yielded different results with a more balanced distribution, however, our study population was representative of the population of Sweden.

Finally, attrition of the study population during long-term follow-up is a common issue in studies with a longitudinal design. The “did not return” cohort can range from a low of 13% to moderate levels of 35% in randomised trials^35–37^. Other follow-up studies experienced dropout rates exceeding 50%^38^, similar to our study’s dropout rate of 58%. Our dropout analysis (Supplemental Table S7) revealed no significant differences in any of the variables. However, it is reasonable to assume that some of the participants who dropped out may have done so because they had no or few issues with their scars. This potentially leads to a selection bias and higher POSAS scores than would have been observed had the original cohort been assessed.

In this study on the long-term scar quality in pediatric patients with burns after skin grafting, we found that younger age, graft site location on the trunk or lower extremity, and longer post-operative healing times, were all associated with higher POSAS scores (indicating worse scar quality) one year after injury. However, the strength of this conclusion is limited by the small study size and the use of photographs for scar assessments.

## Electronic supplementary material

Below is the link to the electronic supplementary material.

Supplementary Material 1

## Data Availability

The dataset used and/or analysed during the current study are available from the corresponding author on reasonable request.
