# Supplementary material for: Evaluating scar outcomes in pediatric burn patients following skin grafting
Source: Sci Rep. 2025 Jun 20;15:20205. doi: 10.1038/s41598-025-06378-y (PMC12181261; doi:10.1038/s41598-025-06378-y)
Supplement: Supplementary file 1 — Supplementary Material 1 [file 41598_2025_6378_MOESM1_ESM.pdf]

Evaluating scar outcomes in pediatric burn patients following skin grafting

Ingrid Steinvall[1]*, Sharon Kennedy[1, 2], Matilda Karlsson[1], Mohamed A. Ellabban[1,3], Folke Sjöberg[1,4],

Caroline Andersson[1], Moustafa Elmasry[1], Islam Abdelrahman[1]

Supplemental Table S1 – Change in POSAS Observer categories over time

Item	Category	3 months	12 months	P value
n		28	23	
Vascularity	Red	13 (46)	0	<0.001
	Pink	14 (50)	17 (74)	0.08
	Pale	0	5 (22)	0.01
	Mix	1 (4)	1 (4)	1.00
n		29	30	
Pigmentation	Hyper	19 (66)	15 (50)	0.23
	Hypo	1 (3)	5 (17)	0.20
	Mix	9 (31)	10 (33)	0.85
n		27	29	
Thickness	Thicker	27 (100)	29 (100)	-
n		29	30	
Relief	More	10 (34)	11 (37)	0.86
	Less	5 (17)	3 (10)	0.47
	Mix	14 (48)	16 (53)	0.70
n		21	22	
Surface area	Contraction	21 (100)	18 (82)	0.11
	Mix	0	4 (18)	0.11

Data are presented as n (%). Chi-squared or Fisher's exact test. The "n" of each item shows that some assessments were done with no recording of category. The percentages are calculated on the available numbers.

Supplemental Table S2 – Details of the patients and POSAS values from the 12 months' follow-up grouped by skin graft operation timing

	Early	Late	p value
Patients	15	15	
Age, years	4.7 (2.3 – 13.0)	2.2 (1.4 – 6.0)	0.35
Male sex	6 (40)	10 (67)	0.14
Skin type I/II/III/IV/V	4/8/3/0/0	1/9/3/1/1	0.43
Burn type:			0.02
Scald	7 (47)	13 (87)	0.02
Contact burn	6 (40)	0	0.02
Flame burn	2 (13)	2 (13)	1.00
Burn size, BSA%	2.5 (0.6 – 10.0)	5.0 (3.0 – 7.0)	0.62
Superficial dermal burn, BSA%	1.0 (0.0 – 5.0)	3.0 (0.0 – 5.5)	0.84
Deep dermal burn, BSA%	0.8 (0.1 – 2.0)	1.0 (0.0 – 2.5)	0.65
Full thickness burn, BSA%	0.0 (0.0 – 0.0)	0.0 (0.0 – 0.0)	0.60
Deep dermal and full thickness burn, BSA%	0.8 (0.3 – 2.0)	1.2 (0.2 – 2.5)	0.57
Operated and skin grafted area, BSA%	1.0 (0.6 – 1.0)	0.8 (0.5 – 1.5)	0.33
Meshed skin graft	8 (53)	12 (80)	0.12
Days from injury to operation	10.0 (6.0 – 12.0)	15.0 (14.0 – 18.0)	<0.001
Patients with two operations	4 (27)	0	0.10
Antibiotic administration (before operation)	6 (40)	10 (67)	0.14
Antibiotic administration (after operation)	7 (47)	5 (33)	0.46
Healing time, days from injury	27.0 (24.0 – 40.0)	28.0 (27.0 – 40.0)	0.62
Days from operation to healing	16.0 (14.0 – 34.0)	13.0 (9.0 – 26.0)	0.02
Vascularity	2.0 (2.0 – 3.0)	1.0 (1.0 – 2.0)	0.02
Pigmentation	3.0 (2.0 – 4.0)	2.0 (2.0 – 3.0)	0.13
Thickness	3.0 (2.0 – 4.0)	2.0 (2.0 – 2.0)	0.01
Relief	3.0 (3.0 – 4.0)	3.0 (2.0 – 4.0)	0.25
Surface area	8.0 (5.0 – 9.0)	7.0 (5.0 – 8.0)	0.71
Score sum	18.0 (16.0 – 23.0)	16.0 (14.0 – 18.0)	0.045

Data are presented as median (25th – 75th centile) and n (%). Mann Whitney U and Chi-squared or Fisher's exact test. Early skin graft operation <14 days after injury, Late skin graft operation $\geq$14 days.

Supplemental Table S3 – Details of the patients and POSAS values from the 12 months' follow-up grouped by meshed and not meshed skin graft

	Meshed	Not meshed	p value
Patients	20	10	
Age, years	2.3 (1.4 – 5.8)	5.4 (4.4 – 14.3)	0.07
Male sex	11 (55)	5 (50)	1.00
Skin type I/II/III/IV/V	2/12/4/1/1	3/5/2/0/0	0.61
Burn type:			0.39
Scald	15 (75)	5 (50)	
Contact burn	3 (15)	3 (30)	
Flame burn	2 (10)	2 (20)	
Burn size, BSA%	5.6 (0.9 – 7.2)	2.8 (0.7 – 7.0)	0.40
Superficial dermal burn, BSA%	3.3 (0.0 – 5.0)	0.3 (0.0 – 7.0)	0.62
Deep dermal burn, BSA%	1.1 (0.1 – 2.4)	0.8 (0.1 – 1.1)	0.42
Full thickness burn, BSA%	0.0 (0.0 – 0.0)	0.0 (0.0 – 0.0)	0.78
Deep dermal and full thickness burn, BSA%	1.4 (0.4 – 2.5)	0.8 (0.2 – 1.1)	0.23
Operated and skin grafted area, BSA%	0.8 (0.5 – 2.3)	0.9 (0.5 – 1.0)	0.75
Operated > 14 days after injury	12 (60)	3 (30)	0.12
Days from injury to operation	14.0 (9.3 – 16.5)	12.0 (9.0 – 14.8)	0.50
Patients with two operations	3 (15)	1 (10)	1.00
Antibiotic administration (before operation)	13 (65)	3 (30)	0.12
Antibiotic administration (after operation)	8 (40)	4 (40)	1.00
Healing time, days from injury	28.0 (24.0 – 39.8)	27.0 (26.0 – 40.3)	0.98
Days from operation to healing	14.0 (9.8 – 27.8)	14.5 (13.8 – 31.3)	0.56
Vascularity	1.5 (1.0 – 2.0)	2.0 (1.8 – 2.3)	0.16
Pigmentation	3.0 (2.0 – 3.0)	2.0 (2.0 – 4.3)	1.00
Thickness	2.0 (2.0 – 3.0)	2.5 (2.0 – 3.3)	0.78
Relief	3.5 (3.0 – 4.0)	3.0 (2.0 – 3.0)	0.049
Surface area	7.0 (6.0 – 8.0)	6.0 (4.0 – 8.3)	0.29
Score sum	17.5 (14.3 – 19.8)	16.5 (12.5 – 19.3)	0.53

Data are presented as median (25th – 75th centile) and n (%). Mann Whitney U and Chi-squared or Fisher's exact test.

Supplemental Table S4 – POSAS items and score sum by skin type

	I	II	III-IV	p
Patients	5	17	8	
Vascularity	2.0 (1.0 – 2.0)	2.0 (1.0 – 2.0)	1.5 (1.0 – 2.8)	0.90
Pigmentation	2.0 (2.0 – 2.5)	2.0 (2.0 – 3.0)	3.0 (2.3 – 3.8)	0.15*
Thickness	2.0 (2.0 – 3.0)	2.0 (2.0 – 3.0)	3.0 (2.0 – 3.8)	0.36
Relief	3.0 (2.0 – 4.0)	3.0 (2.5 – 4.0)	3.0 (3.0 – 3.8)	0.94
Surface area	5.0 (4.0 – 7.5)	7.0 (5.0 – 8.5)	7.5 (6.0 – 8.8)	0.28
Score sum	14.0 (12.0 – 18.0)	17.0 (14.5 – 20.5)	17.5 (16.0 – 19.8)	0.32

POSAS values at 12 months' follow-up are presented as median (25th – 75th centile). Skin type classified according to the Fitzpatrick scale. Kruskal-Wallis ANOVA. *Post hoc Mann-Whitney U test showed p=0.09 for the difference in pigmentation between type I and type III-V.

Supplemental Table S5 – POSAS score sum, median values by body site and a p value matrix

		Trunk	Upper extremity	Lower extremity
	Score sum	p value	p value	p value
Head and neck	16.0 (11.0 – 17.0)	0.10	0.79	0.048
Trunk	19.0 (18.0 – 21.0)		0.047	0.78
Upper extremity	15.5 (13.3 – 17.8)	0.047		0.009
Lower extremity	20.0 (17.3 – 23.8)	0.78	0.009	

POSAS score sum at 12 months follow-up are presented as median (25th – 75th centile). Kruskal-Wallis ANOVA p= 0.02. Post hoc analysis was done with the Mann-Whitney U test, not corrected for repeated analysis.

Supplemental Table S6 – POSAS items and score sum by body site

	Upper extremity	Trunk	Lower extremity	Head and neck	p
Patients	16 (53)	3 (10)	8 (27)	3 (10)	
Vascularity	1.0 (1.0 – 2.0)	2.0 (2.0 – 2.0)	2.0 (1.3 – 3.0)	2.0 (1.0 – 2.0)	0.18
Pigmentation	2.0 (2.0 – 3.0)	2.0 (2.0 – 3.0)	3.5 (3.0 – 4.8)	2.0 (2.0 – 2.0)	0.15
Thickness	2.0 (2.0 – 3.0)	2.0 (2.0 – 3.0)	3.0 (2.3 – 4.5)	2.0 (2.0 – 2.0)	0.14
Relief	3.0 (2.3 – 4.0)	4.0 (4.0 – 4.0)	3.0 (2.3 – 3.8)	3.0 (2.0 – 3.0)	0.16
Surface area	6.5 (4.3 – 7.8)	8.0 (8.0 – 10.0)	9.0 (6.0 – 9.0)	7.0 (4.0 – 8.0)	0.03
Score sum	15.5 (13.3 – 17.8)	19.0 (18.0 – 21.0)	20.0 (17.3 – 23.8)	16.0 (11.0 – 17.0)	0.02

POSAS values at 12 months' follow-up are presented as median (25th – 75th centile). Kruskal-Wallis ANOVA. *Post hoc Mann-Whitney U test showed p=0.02 for the difference in surface between Upper and Lower extremity, and between Upper extremity and Trunk. The post hoc differences in score sum were p=0.009 between Upper and Lower extremity, p=0.047 between Upper extremity and trunk, and p=0.048 between Lower extremity and Head and neck.

Supplemental Table S7 – Dropout analysis, a comparison between the "did not return cohort" and the study group

	Drop outs	Scar follow-up	P value
Patients	45	30	
Age, years	1.9 (1.3 – 9.2)	3.9 (1.5 – 11.6)	0.31
Male sex	28 (62)	16 (53)	0.44
Burn type:			0.69
Scald	26 (58)	20 (67)	
Contact burn	10 (22)	6 (20)	
Flame burn	9 (20)	4 (13)	
Burn size, BSA%	3.0 (1.5 – 9.0)	4.8 (0.8 – 7.1)	0.87
Superficial dermal burn, BSA%	1.5 (0.0 – 3.8)	2.8 (0.0 – 5.1)	0.55
Deep dermal burn, BSA%	1.0 (0.0 – 2.3)	0.9 (0.1 – 2.0)	0.97
Full thickness burn, BSA%	0.0 (0.0 – 0.4)	0.0 (0.0 – 0.0)	0.10
Deep dermal and full thickness burn, BSA%	1.0 (0.3 – 2.5)	1.0 (0.3 – 2.1)	1.00
Operated and skin grafted area, BSA%	1.0 (0.5 – 1.5)	0.8 (0.5 – 1.1)	0.82
Operated > 14 days after injury	22 (49)	15 (50)	0.93
Days from injury to operation	13.0 (7.5 – 18.0)	13.5 (9.8 – 15.5)	0.82
Patients with two operations	4 (9)	4 (13)	0.71
Antibiotic administration (before operation)	23 (51)	16 (53)	0.84
Antibiotic administration (after operation)	22 (49)	12 (40)	0.26
Healing time, days from injury	29.0 (21.5 – 35.5)	28.0 (25.5 – 40.0)	0.26
Days from operation to healing	16.0 (9.0 – 20.5)	14.0 (12.8 – 28.8)	0.31

Data are presented as median (25th – 75th centile) and n (%). Mann Whitney U and Chi-squared tests.
